# Demographic characteristics associated with a penicillin allergy label during pregnancy

**DOI:** 10.3389/falgy.2024.1511392

**Published:** 2024-12-09

**Authors:** Timothy M. Buckey, Patrick K. Gleeson, Cara M. Curley, Scott F. Feldman, Andrea J. Apter, Olajumoke O. Fadugba

**Affiliations:** ^1^ Section of Allergy and Immunology, Division of Pulmonary, Allergy and Critical Care Medicine, Perelman School of Medicine, University of Pennsylvania, Philadelphia, PA, United States; ^2^ Division of Obstetrics and Gynecology, Hospital of the University of Pennsylvania, Philadelphia, PA, United States

**Keywords:** penicillin, antibiotic, allergy, label, demographic, pregnancy, disparity

## Abstract

**Introduction:**

Penicillins and other beta-lactam antibiotics are used in greater than one-third of pregnant women as treatment for Group B Streptococcus colonization and prophylaxis for Caesarean sections. Penicillin allergy labels have been associated with increased morbidity in the pregnant population, and penicillin allergy evaluation during pregnancy is now recognized as safe and effective. Yet, demographic characteristics associated with having a penicillin allergy label during pregnancy have not been studied. We aimed to evaluate factors associated with having a penicillin allergy label in a diverse population of pregnant patients.

**Methods:**

We performed a retrospective observational study of pregnant patients who had an outpatient visit with Obstetrics and Gynecology and a delivery encounter from 1/1/2020 through 6/30/2022 using electronic health record data in a large health system. We used a multivariable logistic regression model to evaluate factors associated with having a penicillin allergy label.

**Results:**

We identified 10,969 pregnant women of whom 940 (8.6%) had a penicillin allergy label. In the multivariable analysis, having a penicillin allergy label was positively associated with age 32−34 years [odds ratio (OR) = 1.31 vs. 18−27 years, *p* = 0.02], 35−51 years (OR = 1.41 vs. 18−27 years, *p* = 0.002) and having rhinitis, asthma, or eczema (OR = 1.55 vs. none, *p* < 0.0005); and negatively associated with Black race (OR = 0.59 vs. White, *p* < 0.0005).

**Discussion:**

This study found that Black race was associated with lower likelihood of penicillin allergy label, while older age and atopic conditions were associated with a higher likelihood. This finding may impact health outcomes and interventions related to penicillin allergy in pregnant women.

## Introduction

A penicillin allergy label is reported in approximately 10% of the population (1, 2). Yet after evaluation, greater than 90% of patients with a reported allergy are able to tolerate penicillins (1, 2). Penicillin and other beta-lactam antibiotics are an essential class of antibiotics during the peripartum and postpartum periods for indications such as Group B *Streptococcus* (GBS) and Cesarean section prophylaxis (3–6). A history of penicillin allergy label in pregnancy is associated with use of second line antibiotics and adverse health outcomes, including increased rates of Cesarean delivery, post-Cesarean wound complications, longer hospitalization, postpartum endometriosis and longer neonatal hospital stay (4, 6). In recent years, the safety and efficacy of penicillin allergy evaluation via skin testing and drug challenge have been investigated, and this evaluation has been increasingly implemented in clinical practice (5, 7, 8). Based on accumulating evidence demonstrating the safety and efficacy of penicillin allergy testing and delabeling in pregnancy, the American College of Obstetrics and Gynecology recommends that individuals with a history of penicillin allergy undergo an evaluation prior to delivery (3).

Patient use of and experiences with healthcare vary based on social and demographic factors (9–11). As drug allergy labels are acquired through healthcare interactions and receipt of antibiotics, different patient experiences with the healthcare system may affect the acquisition of a penicillin allergy label (12). For example, one study showed non-Hispanic Black and Hispanic pediatric patients were less likely to be prescribed antibiotics on presentation to the Emergency Department for viral respiratory infections in comparison to White pediatric patients (13). In addition to antibiotic exposure, the acquisition of an antibiotic allergy label may be associated with health literacy regarding the various types of adverse drug reactions (12).

To date, few studies have evaluated the demographic characteristics associated with having a penicillin allergy label (12, 14–21). Women have been shown to have a higher antibiotic allergy rate compared to men (14, 20, 21), which may be due to the increased exposure to antibiotics among women (20). Older age has been associated with higher antibiotic allergy prevalence (20, 22). This finding may be related to increased antibiotic exposure with age. Among the few studies examining race, some have found that Black people are less likely to have a penicillin allergy compared with White people (15, 17–19), while other studies have found higher prevalence in Black patients (16) or no difference between White and Black patients (14). Notably, none of these studies focused specifically on women or a pregnant population.

Despite the benefits of addressing unverified penicillin allergy labels, a significant portion of eligible patients, particularly pregnant individuals, may not receive the benefit of this evaluation (5, 23). A previous study performed at the University of Pennsylvania Health System (UPHS) demonstrated that among pregnant women referred to Allergy/Immunology clinic for a penicillin allergy evaluation, fewer than half presented for their evaluation (23). Additionally, Black race and Medicaid insurance were strong predictors of not receiving this evaluation (23). While this study identified a key area of inequity in the penicillin allergy evaluation, further investigation is needed to understand potential disparities at other points of the penicillin allergy labeling-delabeling pipeline, including the initial acquisition of a penicillin allergy label.

In this study, we aimed to investigate the characteristics associated with having a penicillin allergy label among a diverse population of pregnant patients in a large health system.

## Methods

We performed a retrospective cohort study of electronic health record (EHR) data within the University of Pennsylvania Health System (UPHS) for encounters dated January 1, 2020 through June 30, 2022. We identified adults (age 18 years or older) who had both a new pregnancy-related Obstetrics and Gynecology (OBGYN) encounter (defined by UPHS encounter visit 1029) and a hospital encounter for delivery. This study received exemption status from the Institutional Review Board of the University of Pennsylvania.

Patient-level data included age (categorized into quantiles based on the age distribution of patients), race (using EHR categories), ethnicity (i.e., Hispanic or Latino), health insurance type (categorized as private/commercial, Medicaid, or Medicare), rhinitis [any form of rhinitis, defined as having any International Classification of Diseases, Tenth Revision (ICD-10) code of J30* or J31*], asthma [defined as having any ICD-10 code of J45*], and eczema (defined as having any ICD-10 code of L20*, L30.8 or L30.9). We combined rhinitis, asthma, or eczema into a composite variable called concomitant atopic disorder.

We used patients' home addresses to calculate the Area Deprivation Index (ADI), a geographic measure of social vulnerability. The ADI was derived by geocoding addresses using the ArcGIS geocoding feature and assigning each geocode to a census block group. We then used 2021 data from the Neighborhood Atlas to assign an ADI to each patient, and categorized ADI into 4 levels (1–25, 26–50, 51–75 and 76–100), in which a higher value indicates greater neighborhood-level deprivation (24, 25).

We collected all allergy data from the dedicated allergy tab in the EHR and identified allergies to oral, intravenous, or intramuscular antibiotics. We categorized these allergy labels into four groups: penicillins, cephalosporins, sulfonamide antibiotics, and all other antibiotics. Using the same EHR-based allergy tab data, we identified whether patients with a penicillin allergy label were delabeled prior to delivery. We excluded patients who had a penicillin allergy that was removed from the EHR prior to the date of the first pregnancy-related OBGYN encounter. Finally, we identified the clinic location for the first pregnancy-related OBGYN encounter (i.e., the 1029 visit) and identified whether patients had any Allergy/Immunology outpatient visits from January 1, 2020 through June 30, 2022.

We evaluated bivariate associations of factors associated with having a penicillin allergy label with age category, race, ethnicity, health insurance type, ADI, rhinitis, asthma, eczema, concomitant atopic disorder, having a cephalosporin allergy label, and having any other antibiotic allergy label using chi-squared tests. To evaluate factors associated with having a penicillin allergy label, we created a multivariable logistic regression model with having a penicillin allergy label as the outcome, and included age, race (categorized as White, Black, or Other due to the small sample sizes for other races), ethnicity, health insurance type, ADI, and having a concomitant atopic disorder as independent variables. Also, we included the clinic location for the earliest pregnancy-related OBGYN encounter as a random intercept in the multivariable model.

## Results

10,969 patients had a pregnancy-related OBGYN encounter, of whom 940 (8.6%) had a penicillin allergy label. Characteristics of these patients are enumerated in Table 1. Patients with a penicillin allergy label were more likely to be 32 years or older (*p* < 0.001 comparing four age quantiles), White (*p* < 0.001 comparing five race categories), have private/commercial health insurance (*p* < 0.001 comparing three health insurance categories), and have an ADI of 50 or under (*p* < 0.001 comparing four ADI groups). Higher rates of rhinitis (*p* < 0.001), asthma (*p* < 0.001), and having a concomitant atopic disorder (*p* < 0.001) were observed in those with a penicillin allergy label. Of the 940 patients with a penicillin allergy label, 51 (5.4%) had a cephalosporin allergy label, 82 (8.7%) had a sulfonamide allergy label, and 132 (14.0%) had another antibiotic allergy label, and each of the three antibiotic allergy label types were more common in patients with a penicillin allergy label than in those without a label (*p* < 0.0001 for all comparisons). Prior to delivery, 298 (31.7%) of patients with a penicillin allergy label had it removed after evaluation of the allergy.

**Table 1:** Patient characteristics related to having a penicillin allergy label among 10,969 pregnant patients.

Factors	Overall *N* = 10,969	No penicillin allergy label *N* = 10,029	Penicillin allergy label *N* = 940	*p*-value
Age in quantiles				
18–27	3,116 (28.4%)	2,930 (29.2%)	186 (19.8%)	<0.001
28–31	2,889 (26.3%)	2,641 (26.3%)	248 (26.4%)	
32–34	2,519 (23.0%)	2,270 (22.6%)	249 (26.5%)	
35–51	2,445 (22.3%)	2,188 (21.8%)	257 (27.3%)	
Race				
White	4,947 (45.1%)	4,362 (43.5%)	585 (62.2%)	<0.001
Black	5,127 (46.7%)	4,811 (48.0%)	316 (33.6%)	
Asian	866 (7.9%)	829 (8.3%)	37 (3.9%)	
American Indian or Alaskan Native	18 (0.2%)	17 (0.2%)	1 (0.1%)	
Native Hawaiian or other Pacific	11 (0.1%)	10 (0.1%)	1 (0.1%)	
Islander				
Ethnicity				
Not Hispanic or Latino	10,366 (94.5%)	9,483 (94.6%)	883 (93.9%)	0.43
Hispanic or Latino	603 (5.5%)	546 (5.4%)	57 (6.1%)	
Insurance				
Private	6,629 (60.4%)	5,970 (59.5%)	659 (70.1%)	<0.001
Medicaid	4,323 (39.4%)	4,046 (40.3%)	277 (29.5%)	
Medicare	17 (0.2%)	13 (0.1%)	4 (0.4%)	
ADI				
1–25	2,618 (23.9%)	2,332 (23.3%)	286 (30.4%)	<0.001
26–50	2,973 (27.1%)	2,672 (26.6%)	301 (32.0%)	
51–75	1,939 (17.7%)	1,791 (17.9%)	148 (15.7%)	
76–100	3,439 (31.4%)	3,234 (32.2%)	205 (21.8%)	
Rhinitis				
No	9,259 (84.4%)	8,539 (85.1%)	720 (76.6%)	<0.001
Yes	1,710 (15.6%)	1,490 (14.9%)	220 (23.4%)	
Asthma				
No	8,551 (78.0%)	7,871 (78.5%)	680 (72.3%)	<0.001
Yes	2,418 (22.0%)	2,158 (21.5%)	260 (27.7%)	
Eczema				
No	10,085 (91.9%)	9,226 (92.0%)	859 (91.4%)	0.18
Yes	884 (8.1%)	803 (8.0%)	81 (8.6%)	
Concomitant atopic disorder				
No rhinitis, eczema, or asthma	7,057 (64.3%)	6,533 (65.1%)	524 (55.7%)	<0.001
Rhinitis, eczema, or asthma	3,912 (35.7%)	3,496 (34.9%)	416 (44.3%)	
Cephalosporin allergy label				
No	10,772 (98.2%)	9,883 (98.5%)	889 (94.6%)	<0.001
Yes	197 (1.8%)	146 (1.5%)	51 (5.4%)	
Sulfonamide allergy label				
No	10,512 (95.8%)	9,654 (96.3%)	858 (91.3%)	<0.001
Yes	457 (4.2%)	375 (3.7%)	82 (8.7%)	
Other antibiotic allergy label				
No	10,443 (95.2%)	9,635 (96.1%)	808 (86.0%)	<0.001
Yes	526 (4.8%)	394 (3.9%)	132 (14.0%)	

Patients without a label (*n* = 10,029) were compared to patients with a label (*n* = 940) using chi-squared tests.

According to the multivariable logistic regression model, having a penicillin allergy label was associated with increasing age for patients aged 32–34 years (OR = 1.31 vs. 18–27 years, *p* = 0.02) and 35–51 years (OR = 1.41 vs. 18–27 years, *p* = 0.002) and with having a concomitant atopic disorder (OR = 1.55, *p* < 0.0005) (Table 2). Compared with White race, Black race (OR = 0.59, *p* < 0.0005) and Other race (OR = 0.36, *p* < 0.0005) were associated with lower odds of having a penicillin allergy label. No associations between having a penicillin allergy label and ADI or insurance were observed.

**Table 2:** Factors associated with having a penicillin allergy label.

Characteristic	Odds ratio (95% CI)	*p*-value
Age in quantiles (18–27 as reference)		
28–31	1.19 (0.97, 1.48)	0.10
32–34	1.31 (1.05, 1.63)	0.02
35–51	1.41 (1.13, 1.75)	0.002
Race (White as reference)		
Black	0.59 (0.48, 0.73)	<0.0005
Other race	0.36 (0.26, 0.50)	<0.0005
Ethnicity (Not Hispanic or Latino as reference)		
Hispanic or Latino	1.02 (0.76, 1.37)	0.89
Insurance (Private insurance as reference)		
Medicaid	0.98 (0.80, 1.21)	0.87
Medicare	3.30 (1.05, 10.37)	0.04
ADI (1–25 as reference)		
26–50	0.98 (0.82, 1.16)	0.80
51–75	0.91 (0.72, 1.15)	0.44
76–100	0.82 (0.63, 1.07)	0.14
Concomitant atopic disorder (No rhinitis, eczema, or asthma as reference)		
Rhinitis, asthma, or eczema	1.55 (1.35, 1.77)	<0.0005
Clinic location for the first pregnancy-related OBGYN encounter*	0.002 (0.000002, 2.83)	0.38

The odds ratios *p*-values were derived from a multivariable logistic regression model among 10,969 pregnant patients.

* Modeled as a random intercept. The *p*-value was computed from a likelihood-ratio test that compared the full model with a model that did not contain this variable.

## Discussion

This study sought to evaluate sociodemographic factors which may impact the upstream portion of the penicillin allergy labeling-delabeling pipeline. An understanding of patient characteristics associated with having a penicillin allergy label can inform conclusions about and subsequent interventions to address drug allergy disparities in the pregnant population. In this cohort, 8.6% of pregnant patients had a penicillin allergy label. This is consistent with previous studies in the pregnant population which have reported penicillin allergy label rates of approximately 8% (4, 6, 26).

Our study found pregnant women of Black race were approximately 40% less likely to have a penicillin allergy label compared to pregnant White women. Other non-White races also had lower likelihood of penicillin allergy label in comparison to those of White race. An association of labeling with ethnicity was not identified, which may be due to the small Hispanic or Latino population in this study. While there are few previous studies evaluating the demographic factors associated with penicillin allergy label, most of these studies found Black individuals, both adults and children, were less likely to have a penicillin allergy compared with White individuals (15, 17–19). Notably, one study evaluating hospitalized adults with skin and soft tissue infections found higher prevalence in Black patients (16). Another study found no difference in penicillin allergy between Black and White patients, but the authors identified a lower prevalence among Asian patients in comparison to White patients (14). The variable findings among these studies likely reflect differences in the population being examined (e.g., pediatric vs. adult, outpatient vs. inpatient) and baseline demographics of the study populations. Our study, for the first time, assessed the demographic differences of penicillin allergy specifically within the female, pregnant population in a large, diverse health system.

Since penicillin allergy is not known to have a genetic basis, it is likely that lower rate of penicillin allergy label acquisition in non-White patients is related to other factors. The acquisition of an antibiotic allergy label requires numerous steps: signs and symptoms of infection, presentation of the patient to the healthcare system, prescription of an antibiotic by a medical provider, development of drug allergy symptoms, patient recognition that they may be experiencing an allergic reaction, reporting of the symptoms by the patient to a healthcare provider, attribution of the reaction to the prescribed antibiotic, communication and documentation of the antibiotic allergy in the patient's medical records, and retention of the antibiotic allergy label by the patient throughout their lifetime (unless or until it is removed).

While our study was unable to examine the details of initial penicillin allergy acquisition, we and others postulate that disparities may occur at one or more points along the aforementioned steps of allergy label acquisition (12, 18). Variable healthcare interactions and literacy regarding the various types of allergic reactions, may explain the differences in the acquisition of a penicillin allergy in pregnant woman and other populations. These findings are particularly interesting in the context of a recent study within our health system demonstrating that among patients who are referred to the Allergy/Immunology clinic for penicillin allergy evaluation, Black race and Medicaid insurance were strong predictors of not receiving this evaluation despite approximately equal numbers of White and Black patients being referred for evaluation (23). In this sense, it appears that different interactions with the healthcare setting may impact the patterns of how penicillin allergy label is both acquired and removed. While a penicillin allergy label is known to confer worse clinical outcomes in pregnancy, future studies should assess if there are variable clinical outcomes of having a penicillin allergy label among individuals of different races during pregnancy (4, 6).

Similar to previous studies, this study found that increasing age is associated with higher likelihood of penicillin allergy label (20, 22). This finding is relevant because older age during pregnancy is associated with higher rates of GBS colonization as well as pregnancy and birth-related complications (27, 28). In our study, individuals with atopic disorders were found to have a higher risk of having a penicillin allergy. Asthma, particularly when uncontrolled, is associated with adverse maternal and fetal health outcomes during pregnancy (29). Therefore, the co-existence of an unverified penicillin allergy label and asthma may confer higher vulnerability to adverse health outcomes in pregnancy.

As knowledge of demographic factors associated with drug allergy label acquisition and evaluation improves, it is also important to examine the patterns and demographic factors that may be associated with referrals for penicillin allergy evaluation, which represents the middle point of the label-delabeling pipeline. In our cohort, only 31.7% of patients had their penicillin allergy delabeled before delivery despite the presence of a penicillin allergy evaluation program for pregnant women at UPHS. There are many potential explanations for this relatively low rate of formal penicillin allergy evaluation and delabeling, which are beyond the scope of this study. Yet if all eligible pregnant patients with a penicillin allergy label are equally referred and present for evaluation, it begets the question: “Do Allergists/Immunologists and health systems have the capacity to evaluate them all before delivery?” Strategies to meet this demand should be developed, including evaluating patients before pregnancy and creating streamlined referral of patients from OBGYN to Allergy Immunology. In health systems where this strategy is not available or feasible, future directions may include non-Allergist assessment of patients with very low risk penicillin allergies.

This study has many notable strengths. It assessed characteristics associated with a penicillin allergy labels in a large cohort of pregnant patients, thus providing new insight on drug allergy in a vulnerable population. This study was performed in Philadelphia where there is a high population of Black individuals (approximately 40%) compared with many other cities, thus allowing a robust analysis of racial disparities (30).

Our study has limitations. We did not review details of the reported penicillin reaction history such as age of labeling (e.g., childhood vs. adult), setting in which labeling occurred, indication for antibiotic use at the time of labeling, and the type or severity of the reaction. This information may have revealed patterns and potential explanations for labeling disparity. This was deemed not practical as it would require individual chart review, and this level of detail is not often documented.

In conclusion, this study identified White race, older age and comorbid atopic disorders as factors associated with a higher prevalence of penicillin allergy labeling in pregnant women. The findings provide new insight to clinicians seeking to address unverified penicillin allergy with the goal of improving clinical outcomes. As strategies are developed to equitably address unverified penicillin allergies in pregnant patients, factors resulting in disparities in the penicillin labeling-delabeling pipeline should continue to be explored and addressed.

## Data Availability

The raw data supporting the conclusions of this article will be made available by the authors, without undue reservation.

## References

[1] Shenoy ES Macy E Rowe T Blumenthal KG. Evaluation and management of penicillin allergy: a review. JAMA. (2019) 321(2):188. 10.1001/jama.2018.19283 30644987

[2] Lee CE Zembower TR Fotis MA Postelnick MJ Greenberger PA Peterson LR The incidence of antimicrobial allergies in hospitalized patients: implications regarding prescribing patterns and emerging bacterial resistance. Arch Intern Med. (2000) 160(18):2819. 10.1001/archinte.160.18.2819 11025792

[3] Prevention of group B streptococcal early-onset disease in newborns: ACOG committee opinion, number 797. Obstet Gynecol. (2020) 135(2):e51–72. 10.1097/AOG.0000000000003668 31977795

[4] Azmy V Lundsberg LS Culhane J Kwah J Partridge C Son M. Pregnant patients with a documented history of penicillin allergy and associated maternal and neonatal outcomes at a tertiary care center. Am J Perinatol. (2024) 41(S 01):e2051–7. 10.1055/a-2096-5002 37211008

[5] Wolfson AR Mancini CM Banerji A Fu X Bryant AS Phadke NA Penicillin allergy assessment in pregnancy: safety and impact on antibiotic use. J Allergy Clin Immunol Pract. (2021) 9(3):1338–46. 10.1016/j.jaip.2020.10.063 33212237 PMC7946747

[6] Desai SH Kaplan MS Chen Q Macy EM. Morbidity in pregnant women associated with unverified penicillin allergies, antibiotic use, and group B Streptococcus infections. Perm J. (2017) 21(1):16–080. 10.7812/TPP/16-080 28333608 PMC5363897

[7] Patel V Gleeson PK Delaney K Ralston SJ Feldman S Fadugba O. Safety and outcomes of penicillin allergy evaluation in pregnant women. Ann Allergy Asthma Immunol. (2022) 128(5):568–74. 10.1016/j.anai.2022.01.032 35123076

[8] Kwah JH Burn MS Liao J Cate J Son M. Outpatient penicillin allergy evaluation during pregnancy and associated clinical outcomes. Am J Obstet Gynecol MFM. (2022) 4(5):100674. 10.1016/j.ajogmf.2022.100674 35691578

[9] Lyratzopoulos G Elliott M Barbiere JM Henderson A Staetsky L Paddison C Understanding ethnic and other socio-demographic differences in patient experience of primary care: evidence from the English general practice patient survey. BMJ Qual Saf. (2012) 21(1):21–9. 10.1136/bmjqs-2011-000088 21900695 PMC3240774

[10] Campbell JL. Age, gender, socioeconomic, and ethnic differences in patients’ assessments of primary health care. Qual Health Care. (2001) 10(2):90–5. 10.1136/qhc.10.2.90 11389317 PMC1757978

[11] Narita RE. Consumption of healthcare services in the United States: the impact of health insurance. J Risk Financ Manag. (2023) 16(5):277. 10.3390/jrfm16050277

[12] Arasaratnam RJ Chow TG Liu AY Khan DA Blumenthal KG Wurcel AG. Penicillin allergy evaluation and health equity: a call to action. J Allergy Clin Immunol Pract. (2023) 11(2):422–8. 10.1016/j.jaip.2022.12.001 36521831

[13] Goyal MK Johnson TJ Chamberlain JM Casper TC Simmons T Alessandrini EA Racial and ethnic differences in antibiotic use for viral illness in emergency departments. Pediatrics. (2017) 140(4):e20170203. 10.1542/peds.2017-0203 28872046 PMC5613999

[14] Albin S Agarwal S. Prevalence and characteristics of reported penicillin allergy in an urban outpatient adult population. Allergy Asthma Proc. (2014) 35(6):489–94. 10.2500/aap.2014.35.3791 25584917 PMC4210656

[15] Zhou L Dhopeshwarkar N Blumenthal KG Goss F Topaz M Slight SP Drug allergies documented in electronic health records of a large healthcare system. Allergy. (2016) 71(9):1305–13. 10.1111/all.12881 26970431 PMC12841114

[16] Wurcel AG Essien UR Ortiz C Fu X Mancini C Zhang Y Variation by race in antibiotics prescribed for hospitalized patients with skin and soft tissue infections. JAMA Netw Open. (2021) 4(12):e2140798. 10.1001/jamanetworkopen.2021.40798 34940871 PMC8703249

[17] Wurcel AG Guardado R Ortiz C Bornmann CR Gillis J Huang K Low frequency of allergy referral for penicillin allergy evaluation in an urban Boston primary care setting. J Allergy Clin Immunol Glob. (2023) 2(1):93–6. 10.1016/j.jacig.2022.09.004 37780102 PMC10509991

[18] Taylor MG Joerger T Li Y Scheurer ME Russo ME Gerber JS Factors associated with penicillin allergy labels in electronic health records of children in 2 large US pediatric primary care networks. JAMA Netw Open. (2022) 5(3):e222117. 10.1001/jamanetworkopen.2022.2117 35285918 PMC9907342

[19] Hampton LL DeBoy JT Hornik CP White MJ Nazareth-Pidgeon KM. Association of sociodemographic factors with reported penicillin allergy in pediatric inpatients. Hosp Pediatr. (2022) 12(7):625–31. 10.1542/hpeds.2021-006462 35660855

[20] Macy E Poon K-Y T. Self-reported antibiotic allergy incidence and prevalence: age and sex effects. Am J Med. (2009) 122(8):778.e1–7. 10.1016/j.amjmed.2009.01.034 19635279

[21] Blumenthal KG Li Y Hsu JT Wolfson AR Berkowitz DN Carballo VA Outcomes from an inpatient beta-lactam allergy guideline across a large US health system. Infect Control Hosp Epidemiol. (2019) 40(05):528–35. 10.1017/ice.2019.50 30915929 PMC6536839

[22] Chiang V Kan AKC Saha C Au EYL Li PH. Identifying the most at-risk age-group and longitudinal trends of drug allergy labeling amongst 7.3 million individuals in Hong Kong. BMC Med. (2024) 22(1):30. 10.1186/s12916-024-03250-0 38273323 PMC10811878

[23] Gleeson PK Rizwan M Apter AJ Katsnelson M Curley CM Fadugba OO. Demographic factors associated with penicillin allergy evaluation in pregnancy. J Allergy Clin Immunol Pract. (2024) 12(2):526–7. 10.1016/j.jaip.2023.11.025 37992893

[24] Kind AJH Buckingham WR. Making neighborhood-disadvantage metrics accessible—the neighborhood atlas. N Engl J Med. (2018) 378(26):2456–8. 10.1056/NEJMp1802313 29949490 PMC6051533

[25] University of Wisconsin School of Medicine and Public Health. Neighborhood Atlas. Area Deprivation Index. [Internet]. Available online at: https://www.neighborhoodatlas.medicine.wisc.edu/ (accessed May 01, 2024).

[26] Macy E. Penicillin skin testing in pregnant women with a history of penicillin allergy and group B streptococcus colonization. Ann Allergy Asthma Immunol. (2006) 97(2):164–8. 10.1016/S1081-1206(10)60007-5 16937745

[27] Khan MA Faiz A Ashshi AM. Maternal colonization of group B streptococcus: prevalence, associated factors and antimicrobial resistance. Ann Saudi Med. (2015) 35(6):423–7. 10.5144/0256-4947.2015.423 26657224 PMC6074477

[28] Cavazos-Rehg PA Krauss MJ Spitznagel EL Bommarito K Madden T Olsen MA Maternal age and risk of labor and delivery complications. Matern Child Health J. (2015) 19(6):1202–11. 10.1007/s10995-014-1624-7 25366100 PMC4418963

[29] Abdullah K Zhu J Gershon A Dell S To T. Effect of asthma exacerbation during pregnancy in women with asthma: a population-based cohort study. Eur Respir J. (2020) 55(2):1901335. 10.1183/13993003.01335-2019 31772000

[30] Philadelphia city, Pennsylvania—Census Bureau Profile [Internet]. Available online at: https://data.census.gov/profile/Philadelphia_city,_Pennsylvania?g=160XX00US4260000 (accessed July 12, 2024).

